# Mechanosensitive ion channels MSL8, MSL9, and MSL10 have environmentally sensitive intrinsically disordered regions with distinct biophysical characteristics in vitro

**DOI:** 10.1002/pld3.515

**Published:** 2023-08-03

**Authors:** Aidan J. Flynn, Kari Miller, Jennette M. Codjoe, Matthew R. King, Elizabeth S. Haswell

**Affiliations:** ^1^ Department of Biology Washington University in St. Louis St. Louis Missouri USA; ^2^ NSF Center for Engineering Mechanobiology, Department of Biology Washington University in St. Louis St. Louis Missouri USA; ^3^ Department of Biochemistry and Biophysics Washington University in St. Louis St. Louis Missouri USA; ^4^ Department of Biomedical Engineering Washington University in St. Louis St. Louis Missouri USA

**Keywords:** *Arabidopsis thaliana*, circular dichroism, intrinsically disordered protein, ion channel, mechanobiology, phase separation, transmembrane protein

## Abstract

Intrinsically disordered protein regions (IDRs) are highly dynamic sequences that rapidly sample a collection of conformations over time. In the past several decades, IDRs have emerged as a major component of many proteomes, comprising ~30% of all eukaryotic protein sequences. Proteins with IDRs function in a wide range of biological pathways and are notably enriched in signaling cascades that respond to environmental stresses. Here, we identify and characterize intrinsic disorder in the soluble cytoplasmic N‐terminal domains of MSL8, MSL9, and MSL10, three members of the MscS‐like (MSL) family of mechanosensitive ion channels. In plants, MSL channels are proposed to mediate cell and organelle osmotic homeostasis. Bioinformatic tools unanimously predicted that the cytosolic N‐termini of MSL channels are intrinsically disordered. We examined the N‐terminus of MSL10 (MSL10^N^) as an exemplar of these IDRs and circular dichroism spectroscopy confirms its disorder. MSL10^N^ adopted a predominately helical structure when exposed to the helix‐inducing compound trifluoroethanol (TFE). Furthermore, in the presence of molecular crowding agents, MSL10^N^ underwent structural changes and exhibited alterations to its homotypic interaction favorability. Lastly, interrogations of collective behavior via in vitro imaging of condensates indicated that MSL8^N^, MSL9^N^, and MSL10^N^ have sharply differing propensities for self‐assembly into condensates, both inherently and in response to salt, temperature, and molecular crowding. Taken together, these data establish the N‐termini of MSL channels as intrinsically disordered regions with distinct biophysical properties and the potential to respond uniquely to changes in their physiochemical environment.

## 1 INTRODUCTION

In recent years, the function of intrinsically disordered regions (IDRs) in plant biology has become an attractive topic due to their putative roles in a range of functions, including transcription, scaffolding, and stress response (Covarrubias et al., 2017; Emenecker et al., 2020). IDRs are protein regions that lack stable secondary structures, instead dynamically sampling a series of conformations over time known as a conformational ensemble (Dunker et al., 2013). These conformational ensembles can dictate the biological function of the disordered region and are often influenced by the surrounding cellular context (Cohan et al., 2019; Das & Pappu, 2013; Mao et al., 2012). Due to this environmental sensitivity, IDRs are proposed to provide a molecular mechanism for sensing and responding to intracellular stress (Cuevas‐Velazquez & Dinneny, 2018; Emenecker et al., 2020). Further, natural IDRs have been repurposed as environmental sensors in experimental settings (Cuevas‐Velazquez et al., 2021).

IDRs localize to a variety of different cellular contexts and compartments. Yet, only a fraction of IDRs present at membranes have been experimentally characterized in detail (Goretzki et al., 2021; Kjaergaard & Kragelund, 2017; Verkest et al., 2022), despite a significant enrichment of IDRs in cytosolic extensions of membrane proteins compared to the full proteome (Bürgi et al., 2016). Due to the established ability of IDRs to sense specific environmental cues, and their proximity to the site of physical or chemical recognition (the membrane), membrane‐tethered IDRs are commonly used by cells to rapidly recognize and respond to stimuli (van der Lee et al., 2014). In such responses, IDR‐rich cytoplasmic domains can drive the assembly of biomolecular condensates through an associative transition that may or may not be coupled to a segregative phase transition (Mittag & Pappu, 2022; Zhao & Zhang, 2020). Associative transitions are driven by interactions among so‐called stickers, which can constitute folded domains, short motifs, or even single amino acid residues. Segregative transitions, such as classical liquid–liquid phase separation (LLPS), are driven by a solubility mismatch between so‐called spacers—regions that separate stickers—and the surrounding milieu (Choi et al., 2020; Martin & Mittag, 2018). The complex interplay of associative and segregative transitions within a system leads to biomolecular condensates that exhibit a range of morphologies and material properties (Martin et al., 2020; Mittag & Pappu, 2022).

Mechanosensitive (MS) ion channels are transmembrane channels that open and allow ion flux in response to mechanical force applied to the membrane in which they are embedded. In plants, MS ion channels are implicated in the perception and response to a wide variety of stimuli, including germination, cell wounding, and osmotic stress (Basu & Haswell, 2017). In the model flowering plant *A. thaliana*, there are 10 members of the MscS‐like (MSL) protein family that share homology with the *Escherichia coli* MS channel MscS (Mechanosensitive channel of Small conductance) (Haswell, 2007; Pivetti et al., 2003). The ten MSL channels in Arabidopsis have different expression profiles and localize to various organellar membranes—including the mitochondrial, plastidial, and plasma membranes (Haswell, 2007; Haswell et al., 2008; Haswell & Meyerowitz, 2006; Lee et al., 2016).

MSL channels are generally thought to act as “osmotic safety valves” that prevent organelle or cell bursting by opening in response to increased membrane tension during hypo‐osmotically induced swelling (Basu & Haswell, 2017). However, additional functions and modes of regulation have been proposed (Veley et al., 2014; Wang et al., 2021). For example, MSL10 promotes programmed cell death when overexpressed or in response to cell swelling in seedlings (Basu & Haswell, 2020; Basu et al., 2020; Veley van der Lee et al., 2014), perhaps as a response to pathogenic invasion (Basu et al., 2022). This activity is ablated by phosphomimetic mutations at seven phosphorylation sites in the soluble 164‐residue N‐terminus of MSL10 (Basu et al., 2020; Basu & Haswell, 2020; Veley et al., 2014). The cell death‐promoting activity of MSL10 is both physically and genetically separable from its function as a mechanosensitive ion channel (Maksaev et al., 2018).

MSL10 is localized to the plasma membrane and expressed ubiquitously throughout the plant (Haswell et al., 2008). The channel has a slight preference for anions but is otherwise non‐selective (Haswell et al., 2008; Maksaev & Haswell, 2012). MSL9, a close homolog of MSL10, is expressed in root tips and has no known genetic function. MSL9 and MSL10 likely form a mixture of homomeric and heteromeric complexes in vivo (Haswell et al., 2008). A third member of the MSL channel family, MSL8, is expressed exclusively in pollen, where it is required for normal survival of pollen grain rehydration and pollen tube growth (Hamilton et al., 2015; Wang et al., 2021). Here, we provide an analysis of intrinsic disorder in the soluble N‐termini of MSL8, MSL9, and MSL10, using both in silico and in vitro approaches. We find that these regions are predicted to be intrinsically disordered and demonstrate that this disorder is likely ubiquitous throughout putative orthologs of MSL9 and MSL10 in angiosperms. Our work establishes the presence of IDRs in MSL channels and implicates their relevance in MSL channel functions. In addition, these data provide a new collection of evolutionarily related IDRs with notably different environmental responses for future study.

## 2 RESULTS

### 2.1 The N‐termini of MSL8, MSL9, and MSL10 have amino acid compositions characteristic of intrinsically disordered regions and are predicted to be disordered

**FIGURE 1:**
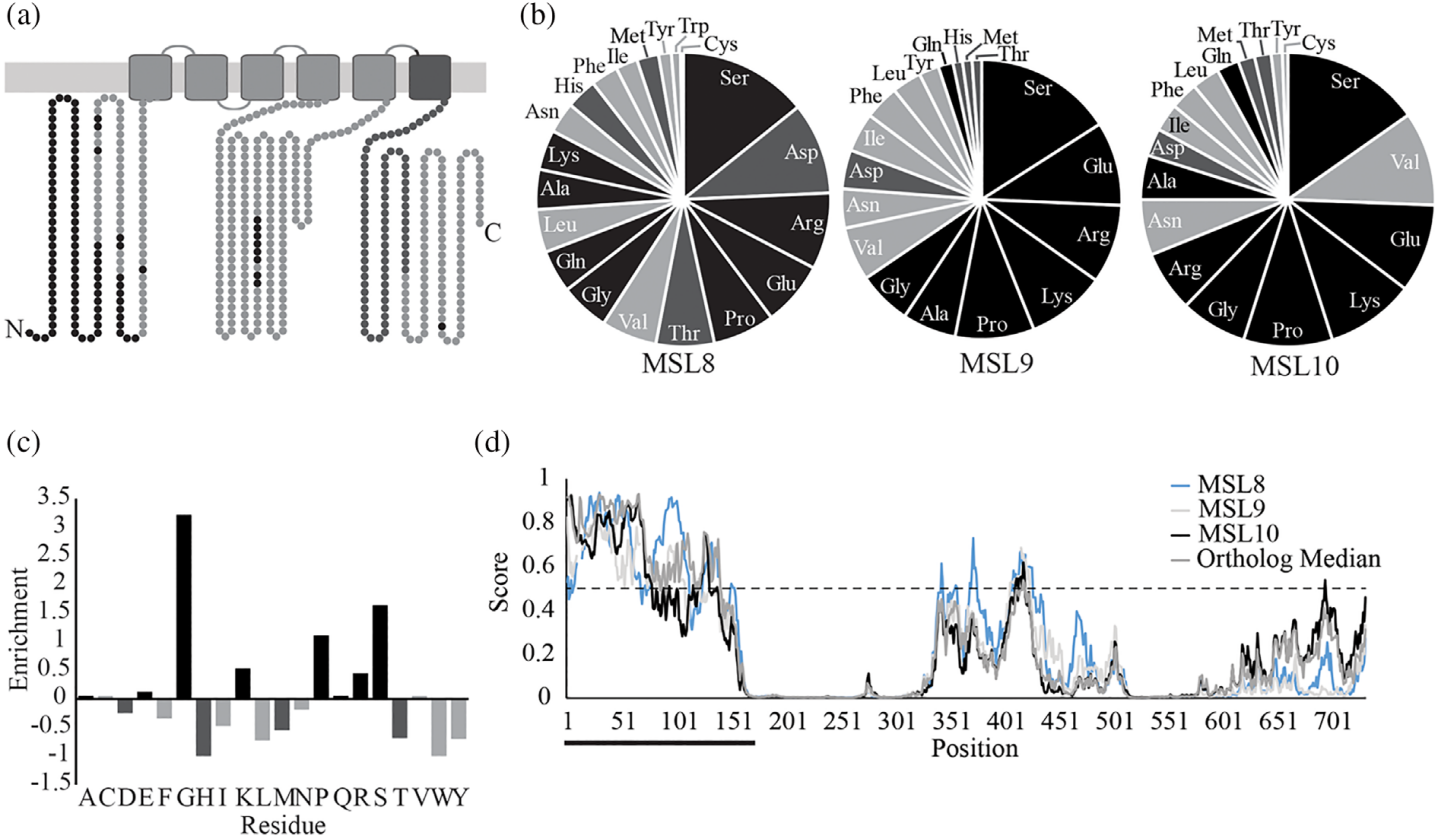
**The N‐termini of MSL family proteins are predicted to be disordered.** (a) Topology and predicted disorder for MSL10. MSL8 and MSL9 present similar topologies. Residues predicted by IUPRED2A to be disordered are shown in black. The conserved MscS domain of MSL10 is dark gray. (b) Amino acid composition of the N‐termini of MSL8, MSL9, and MSL10. Calculations were performed using the PredictProtein webserver. Disorder‐promoting, order‐promoting, and neutral residues are shown in black, light gray, and dark gray, respectively. (c) Compositional profile of the MSL10 N‐terminus compared to the MSL10 C‐terminus (aa 562–734). Comparisons were performed using the composition profiler webtool. Enrichment of a particular amino acid is calculated as (composition N‐terminus–composition C‐terminus)/composition C‐terminus, where the composition is the fractional amount of an amino acid within the N‐ or C‐terminus. Color of the bars as in (b). (d) IUPRED2A disorder profiled for full‐length MSL8, MSL9, MSL10, and the median value of selected orthologs at a given aligned position of MSL10. Residues with a disorder score higher than .5 are predicted to be part of a disordered region, indicated by the dashed line. The black bar indicates N‐terminal regions.

Of the 10 MSL channels in Arabidopsis, MSL8, MSL9, and MSL10 are all localized to the plasma membrane and all three have the same predicted topology (Haswell, 2007). They are comprised of a soluble N‐terminal domain of approximately 150 to 300 aa, six transmembrane helices including the conserved pore‐lining helix 6, a soluble domain between TM helices 4 and 5, and a soluble C‐terminal domain that includes the MscS homology domain (Figure 1a). Outside of the pore‐lining and transmembrane domains, there is low sequence conservation between MSL8, MSL9, and MSL10, especially in the soluble N‐ terminal domain (Figure S1). As evolutionarily related IDRs often exhibit high sequence variability (Wallmann & Kesten, 2020; Zarin et al., 2017), this lack of conservation led us to hypothesize that the N‐termini of MSL channels are intrinsically disordered.

Many IDRs are defined by characteristic amino acid compositions such as a depletion of hydrophobic residues that drive protein folding events and enrichment in hydrophilic, charged, and polar residues (Uversky, 2013, 2019; van der Lee et al., 2014; Yachdav et al., 2014). For instance, IDRs in the DisProt database contain on average ~60% disorder‐promoting residues with notably high percentages of proline (~8%), glutamate (10%), serine (~9%), and lysine (~8%) (Uversky, 2015). We therefore assessed the amino acid frequencies present in the soluble N‐termini of MSL8, MSL9, and MSL10. As shown in Figure 1b, the N‐termini of MSL8 (amino acids 1–296, hereafter referred to as MSL8^N^), MSL9 (amino acids 1–176, hereafter referred to as MSL9^N^), and MSL10 (amino acids 1–164, hereafter referred to as MSL10^N^) contain 46%–58% disorder‐promoting residues like serine, glutamate, arginine, proline, and glycine and only 35%–37% order‐promoting residues like cysteine, tyrosine, isoleucine, tryptophan, and phenylalanine. Furthermore, MSL10^N^ is enriched in residues associated with disorder and depleted in order‐promoting residues relative to the MSL10 C‐terminus according to the Composition Profiler webtool (Vacic et al., 2007) (Figure 1c). Collectively, these results demonstrate that the N‐termini of MSL8, MSL9, and MSL10 have sequence characteristics commonly associated with IDRs.

We next employed IUPRED2A (Mészáros et al., 2018) to predict disordered regions in MSL8, MSL9, and MSL10 (Figure 1d). MSL8^N^, MSL9^N^, and MSL10^N^ were predicted to be disordered, with MSL8^N^ having the highest propensity for disorder. The cytoplasmic loop (amino acids 306–512 of aligned profiles) was predicted to be partially disordered for all three MSL channels examined. Four other webserver‐based algorithms (PONDR‐VLXT, SPOT‐Disorder2, PrDOS, and MFDp2) made similar predictions of strong disorder in the N‐terminus and modest disorder in the cytoplasmic loop of MSL10 (Figure S2a–d). Disordered regions are often highly variable in terms of primary sequence; however, the structural character of disorder within an IDR is sometimes highly conserved (Wallmann & Kesten, 2020). Our above findings suggested that intrinsic disorder is such a conserved feature within Arabidopsis MSL channels. We therefore aligned the amino acid sequence of 13 putative orthologs of MSL9 and MSL10 from monocots and dicots (as reported in Basu et al., 2020) and computed the median of their disorder scores at each position aligned with MSL10 (Christensen et al., 2019) (Figures S2e and 1d). The median score predictions closely matched the profiles of *A. thaliana* MSL9 and MSL10. The data shown in Figures 1, S1, and S2 cumulatively suggest that intrinsic disorder is an evolutionarily conserved property of the N‐termini of plasma‐membrane localized MSL channels in plants.

### 2.2 The N‐terminus of MSL10 is disordered in vitro and self‐associates at high temperatures

**FIGURE 2:**
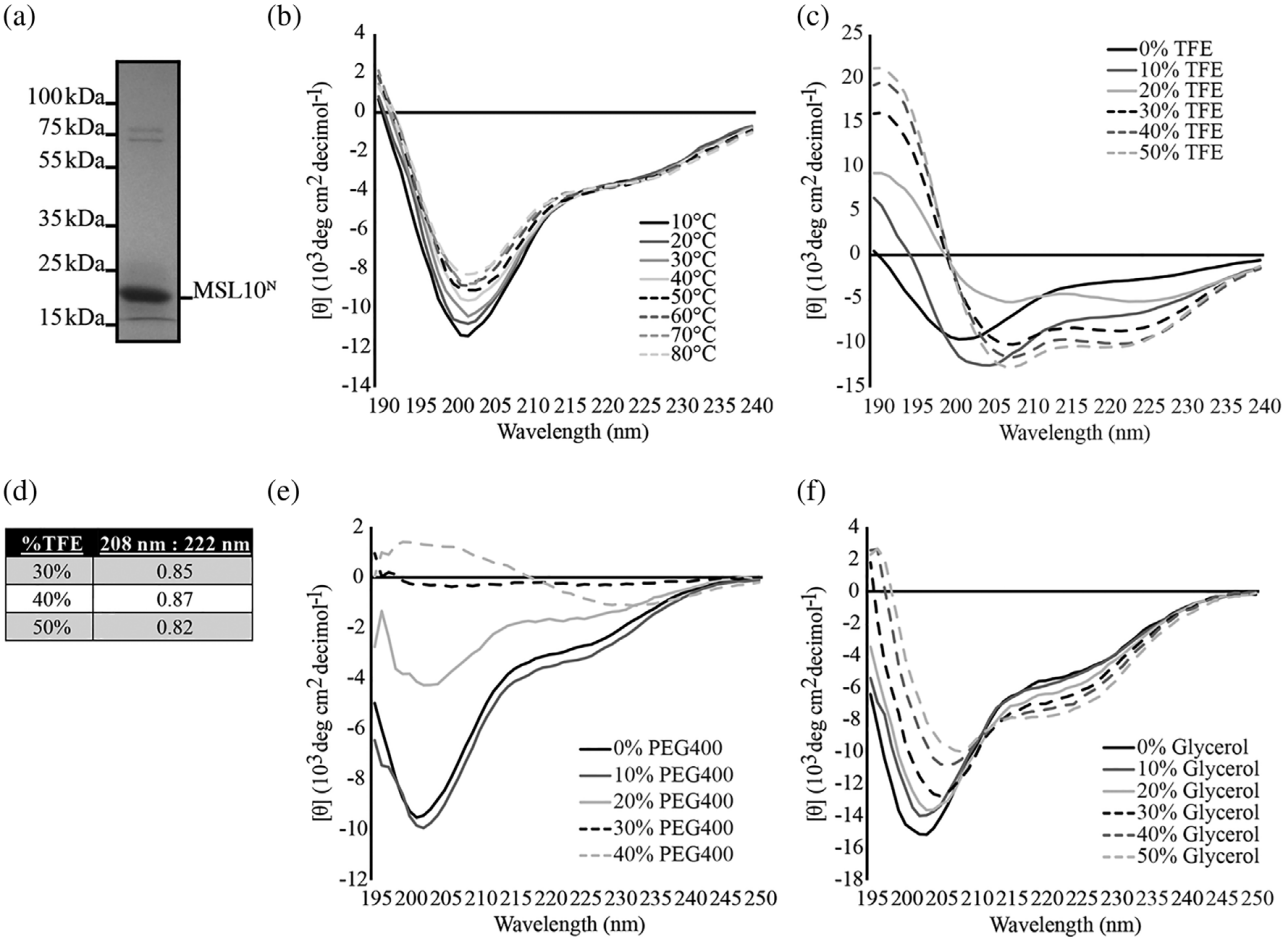
**The MSL10 N‐terminus is disordered and structurally responds to a variety of environments in vitro.** (a) Representative coomassie‐stained 10% sodium dodecyl sulphate‐polyacrylamide gel electrophoresis (SDS‐PAGE) gel of his‐tagged MSL10^N^. His‐tagged MSL10^N^ has an approximate molecular weight of 19 kDa. Circular dichroism spectra of his‐tagged MSL10^N^ when exposed to increasing temperatures (b), TFE (c), PEG 400 (e), and glycerol (f). Spectra were obtained at 20°C in 20 mM sodium phosphate buffer, pH 7.4 unless otherwise specified. (d) The ratio of the mean residue ellipticity value at 222 and 208 nm for the indicated TFE treatments.

To test this prediction experimentally, we focused on MSL10, as its N‐terminal domain has known functions in vivo (Basu et al., 2020; Maksaev et al., 2018; Veley et al., 2014). We purified recombinant C‐terminally His‐tagged MSL10^N^ (Figure 2a) and performed far‐UV circular dichroism (CD) spectroscopy. Measured spectra at 20°C presented a minimum near 200 nm (202 nm), which is typical of disordered proteins (Na et al., 2018) (Figure 2b). The slight shoulder visible at 222 nm is indicative of residual helical structure (Greenfield, 2006). Under increasing temperature, the minimum at 202 nm gradually reduced in magnitude but did not shift left or right. There were no substantial changes seen in the HT voltages associated with CD spectra captured at each temperature (Figure S3a). This collective behavior is associated with an increase in homotypic interactions between monomers (Urry & Ji, 1968). Thus, the N‐terminus of MSL10 is largely disordered and may undergo a modest increase in homotypic interactions with elevated temperature.

### 2.3 The N‐terminus of MSL10 undergoes structural changes when exposed to helical‐inducing compounds in vitro

The N‐terminus of MSL10 has a relatively low fraction of charged residues, resulting in its classification as a Janus sequence on the Das‐Pappu plot (Figure S3b). Some IDRs that fall under this classification may take on folded or disordered ensembles depending on environmental contexts (Das et al., 2015; Das & Pappu, 2013). For instance, previous work has demonstrated that some IDRs transition to a helical conformation upon binding specific partners (Glover et al., 2016; Moritsugu et al., 2012). To further examine the potential of MSL10^N^ to participate in interactions that cause disorder‐to‐order transitions, we introduced 2,2,2‐Triflouroethanol (TFE) to His‐tagged MSL10^N^ and measured structural changes via CD. TFE induces structure in a subset of disordered regions that have a susceptibility to forming alpha‐helices, such as those that fold upon protein interaction (Chemes et al., 2012; Hua et al., 1998; Luo & Baldwin, 1997). As shown in Figure 2c, the addition of TFE resulted in a loss of the spectral minimum at 202 nm and the establishment of two minima at approximately 208 and 222 nm. This profile is characteristic of helical proteins (Greenfield, 2006). For MSL10^N^ in 30%–50% TFE, the ratio of values at 222 to 208 nm was less than .9 (Figure 2d), signifying that the acquired helicity of MSL10^N^ occurs in an isolated helix form, as opposed to multimers of helices (e.g., coiled coils), which are associated with ratiometric values greater than 1.10 (Lau et al., 1984). Thus, the MSL10 N‐terminus is capable of folding in the presence of TFE in vitro.

### 2.4 MSL10's N‐terminus is sensitive to molecular crowding in vitro

Previous in vitro studies have demonstrated changes in some IDR ensembles in response to molecular crowders and small molecules. For instance, some plant IDRs such as members of the late embryogenesis abundant (LEA) families take on a helical structure and undergo increased self‐association in the presence of these components (Chemes et al., 2012; Dorone et al., 2021; Mansouri et al., 2016; Rivera‐Najera et al., 2014). We therefore conducted CD with His‐tagged MSL10^N^ in the presence of polyethylene glycol 400 (PEG 400) and glycerol, both of which simulate crowding (Cuevas‐Velazquez et al., 2016; Nakano et al., 2014; Rivera‐Najera et al., 2014). In response to 10% and 20% PEG 400, the disordered minimum at 202 nm shifted rightward (Figure 2e). At 20% PEG 400, a significant flattening of the spectrum was also observed. Measurements in 30% PEG 400 resulted in near‐zero absorbances, whereas 40% PEG 400 caused the appearance of a small minimum at 232 nm and the disappearance of all other spectrum characteristics. These changes are consistent with increased self‐association with increasing quantities of PEG 400 (Urry et al., 1970; Urry & Ji, 1968).

The addition of glycerol resulted in the appearance of a more defined minimum at 222 nm and the shifting of the disordered minimum at 202–208 nm (Figure 2f), indicating increased helicity (Greenfield, 2006). The presence of an isodichroic point—a position where all spectra intersect—near 212 nm suggests that transitioning from a predominately disordered state to a helical structure is a reversible two‐state process. These results are consistent with a model wherein increasing amounts of glycerol promote the folding of MSL10^N^ into a largely helical conformation. Collectively, the data shown in Figure 2 demonstrate that the disordered MSL10^N^ retains the ability to fold and/or self‐associate in various solution environments.

### 2.5 Phosphomimic and phosphodead mutations do not affect the folding of MSL10^N^

**FIGURE 3:**
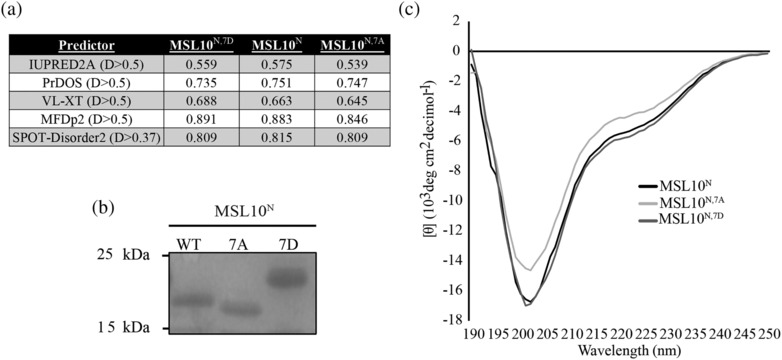
**Neither phosphomimetic nor phosphodead mutations affect the folding of MSL10** ^**N**^. (a) Individual disorder scores for each amino acid were averaged for wild‐type, phosphomimetic, and phosphodead variants of MSL10^N^ using the indicated prediction algorithms. Binary disorder–order cutoffs for each prediction program are shown in parentheses. (b) Coomassie‐stained 10% sodium dodecyl sulphate‐polyacrylamide gel electrophoresis (SDS‐PAGE) gel. (c) Circular dichroism spectra of the indicated variants, obtained at 20°C in 20 mM sodium phosphate buffer, pH 7.4.

Some IDRs undergo folding events in response to changes in the phosphorylation state, thereby regulating protein–protein interactions (Bah et al., 2015). As phosphomimetic lesions in the MSL10 N‐terminus lead to the inactivation of the MSL10 cell death signaling pathways (Veley et al., 2014), we investigated whether these same mutations also affect folding. We first compared the average per residue disorder scores of wild‐type MSL10^N^, phosphodead (MSL10^N, 7A^), and phosphomimetic (MSL10^N, 7D^) sequences from five separate disorder predictors (Figure 3a). Three out of five predictors showed that phosphomimetic substitutions resulted in a .75%–2.5% increase in propensity for disorder, whereas phosphodead substitutions decreased disordered propensity by .65–3.7%, as compared to wild‐type MSL10^N^. To determine whether these substitutions affect the structure of the MSL10 N‐terminus experimentally, we purified His‐tagged MSL10^N^, MSL10^N,7A^, and MSL10^N,7D^. These variants had different mobility in sodium dodecyl sulphate‐polyacrylamide gel electrophoresis (SDS‐PAGE) (Figure 3b), perhaps due to changes in net charge. The CD spectra recorded for MSL10^N^ variants were all highly similar, implying that the N‐terminus is largely disordered regardless of the phosphorylation state (Figure 3c).

### 2.6 Self‐association and phase separation of MSL9^N^ and MSL10^N^

**FIGURE 4:**
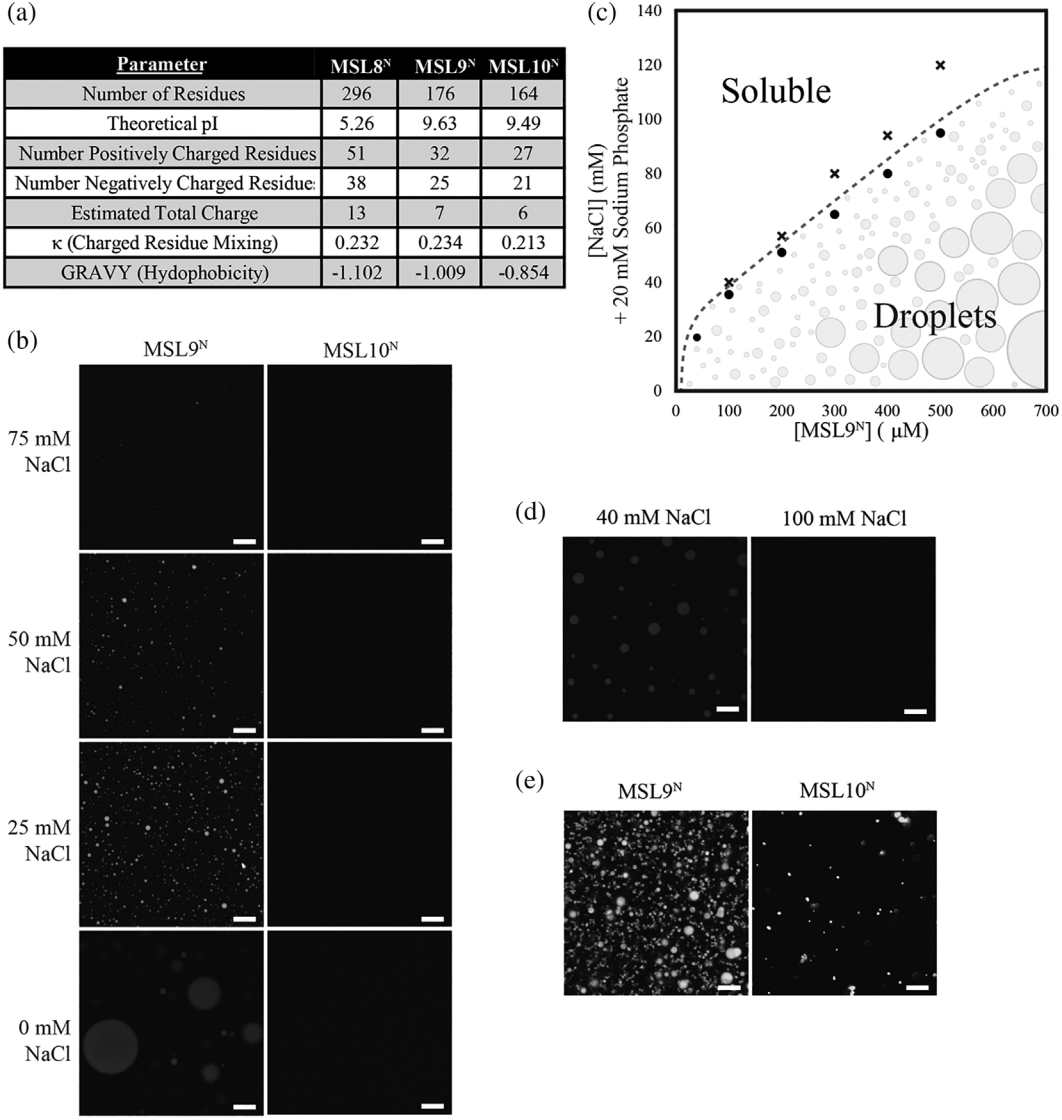
**Self‐assembly behavior of MSL9** ^**N**^ **and MSL10** ^**N**^. (a) Key parameters of MSL8^N^, MSL9^N^, and MSL10^N^ protein sequences as calculated by the ProtParam webtool. (b) Fluorescence images of C‐terminally his‐tagged MSL9^N^ and MSL10^N^ in different salt concentrations at ~500 μM protein. (c) Phase diagram of MSL9^N^ as a function of protein and NaCl concentration. Black crosses denote tested conditions at which condensates were absent, whereas black circles are conditions that produced condensates. (d) Fluorescence images of MSL9^N^ wherein protein was treated with additional salt after droplet formation. Protein concentration before and after salt addition was ~550 μM. (e) Images of his‐tagged MSL9^N^ and MSL10^N^ in response to 30% PEG 400 treatment at ~500 μM protein. In b, d, and e, scale bars are 10 mm.

Many IDRs form networks of weak, multivalent associative interactions that result in the formation of biomolecular condensates (Feng et al., 2019). Furthermore, condensates have been implicated in a range of plant stress responses such as heat, salt, and water stress response (Cuevas‐Velazquez & Dinneny, 2018; Dorone et al., 2021; Emenecker et al., 2020). Although poorly conserved on a sequence level (Figure S1), MSL9^N^ and MSL10^N^ exhibit similarities in charge segregation, hydrophobicity, and patterning of hydrophobic (aromatic) charged blocks along the sequence (Figure 4a). Such sequence characteristics are often found in IDRs that form condensates (Emenecker et al., 2020). Due to these sequence biases and to the observed ability of MSL10^N^ to self‐associate in our CD assays, we probed MSL9^N^ and MSL10^N^ for their ability to form condensates in vitro.

We first imaged the purified N‐termini at various protein (42–1000 μM) and NaCl (0–200 mM) concentrations as described in (Alberti et al., 2018). MSL10^N^ did not form condensates or any visible higher order assemblies in 0–200 mM NaCl at 500 μM protein (Figure 4b, right panels). At this same protein concentration, MSL9^N^ self‐assembled into condensates at NaCl concentrations under 75 mM (Figure 4b, left panels). MSL9 ^N^ condensates were spherical in shape and their size ranged from ~1 to 20 μM in diameter. A partial MSL9^N^ phase diagram that further details the conditions at which MSL9^N^ forms condensates is depicted with an approximate phase boundary in Figure 4c.

Condensate formation is often driven in part by the segregative transition of LLPS, with the resultant condensates exhibiting several features including spherical shape and reversibility of self‐assembly (Alberti & Hyman, 2021). To determine if MSL9^N^ condensates also exhibit the latter property of condensates, we placed MSL9^N^ (550 μM) in 40 mM NaCl to induce the formation of condensates before adding MSL9^N^ stored in high‐salt conditions to achieve a final concentration of 100 mM NaCl without changing protein concentration. Upon exposure to 100 mM NaCl, preformed MSL9^N^ condensates dispersed spontaneously, indicating that MSL9^N^ condensates are reversible (Figure 4d). This, in combination with the spherical nature of these condensates, provides evidence that MSL9^N^ condensates are liquid‐like.

Due to our observations of MSL10^N^ self‐association in response to PEG 400 (Figure 2e), we assessed self‐assembly following PEG treatment. In the presence of 30% PEG 400, MSL10^N^ formed assemblies that ranged from ~1–3 μM in size and consisted of small, spherical condensates (Figure 4e). This finding further implies that increased molecular crowing may drive MSL10^N^ assembly, which is consistent with numerous studies of IDRs under crowding conditions and the expectations of polymer physics theory (Brangwynne et al., 2015; Choi et al., 2020). The addition of 30% PEG 400 to MSL9^N^ samples resulted in smaller yet still spherical condensates (~1–5 μM in diameter) than those seen in phosphate buffer alone at the same protein concentration (compare Figures 4b and e). The clustering of MSL10^N^ assemblies and the reduced size of MSL9^N^ condensates aligns with experimental observations and the theory that condensate morphology is dependent on solution conditions (Boeynaems et al., 2019; Ruff et al., 2019; Zhang et al., 2015). In this case, the protein and condensate dynamics appear to be constrained by crowding (Emenecker et al., 2021; Linsenmeier et al., 2022).

### 2.7 Self‐associative and phase separation behavior of MSL8^N^

**FIGURE 5:**
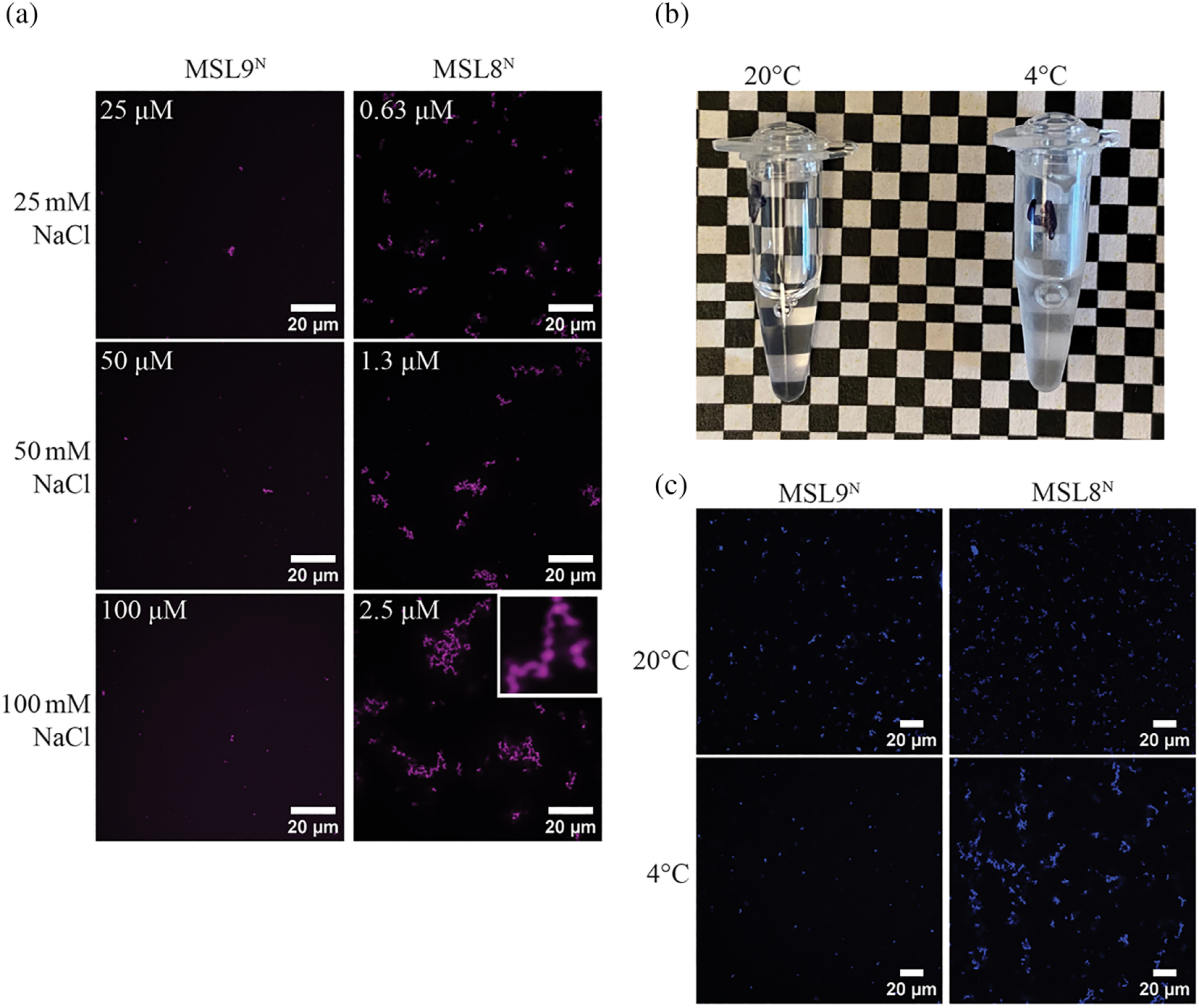
**MSL8** ^**N**^ **forms condensates in response to salt and to low temperatures.** (a) Fluorescence images of his‐tagged MSL8^N^ and MSL9^N^ with increasing levels of salt in 20 mM sodium phosphate. Protein concentration is in the upper left side of each image. (b) Image of tubes containing purified MSL8^N^ at the indicated temperatures. (c) Fluorescence images of MSL9^N^ and MSL8^N^ at room temperature (upper) and 4°C (lower). The samples in these images are in 50 mM NaCl, 20 mM sodium phosphate. The protein concentrations of MSL9^N^ and MSL8^N^ preparations were approximately 10 and 2.5 μM, respectively.

Although MSL8^N^ exhibits some similar sequence characteristics to MSL9^N^ and MSL10^N^ such as charged residue mixing (Figure 4a), MSL8^N^ demonstrates a large expansion of the N‐terminal sequence as well as the acquisition of different sequence characteristics like the number of charged residues (Figure S4). Therefore, we investigated purified MSL8^N^ for its ability to self‐associate. We found that MSL8^N^ formed chain‐like clustered assemblies consisting of small spherical condensates, which is generally indicative of slower condensate dynamics (Emenecker et al., 2021; Linsenmeier et al., 2022) (Figure 5a). The observed MSL8^N^ assemblies resembled condensates observed for MSL10^N^ with higher molecular crowding (Figure 4e). Furthermore, we noted that cold temperatures (4°C) caused MSL8^N^ protein in a storage solution to become turbid, whereas a room temperature replicate remained clear (Figure 5b). All phase‐separated systems that are not arrested, including those comprised of solely IDRs, undergo changes in condensation as temperature changes (Mittag & Pappu, 2022). Since the directions of these changes are informative of the underlying condensation mechanism, we assessed how temperature affected MSL8^N^ and MSL9^N^ self‐assembly. We observed larger clustered assemblies of MSL8^N^ in cold temperatures compared to room temperature. In contrast, MSL9^N^ produced fewer or smaller assemblies when exposed to cold temperatures (Figure 5c). Collectively, our findings highlight that MSL8^N^, MSL9^N^, and MSL10^N^ form condensates, albeit in response to strikingly different environmental conditions.

## 3 DISCUSSION

### 3.1 The soluble N‐termini of MSL8, MSL9, and MSL10 are environmentally sensitive intrinsically disordered regions in vitro

Although the study of intrinsically disordered protein regions has grown enormously in recent years, few studies address IDRs tethered to membrane proteins such as ion channels (Csizmadia et al., 2020). Here, we document the presence of IDRs in the soluble N‐terminus of *A. thaliana* mechanosensitive ion channels and characterize their properties, adding to our understanding of IDR properties found at the membrane.

Although the primary sequences of MSL channel N‐termini are poorly conserved, disorder appears to be a trait that was maintained throughout evolutionary history. In contrast to folded domains, the sequences of disordered regions are typically functionally driven by bulk sequence features such as the amino acid composition, charge, or presence of disorder in the region. Thus, their primary sequence (i.e., the order of amino acids) can undergo large perturbations without affecting the composition and consequently IDR function (Cohan et al., 2022; Zarin et al., 2017). Indeed, multiple algorithms predicted intrinsic disorder in the soluble N‐termini of MSL8, MSL9, and MSL10—and in the same domain of MSL orthologs throughout the plant lineage (Figures 1 and S2).

These predictions were born out in CD experiments with purified recombinant protein of the exemplar MSL10^N^ (Figure 2). MSL10^N^ spectra exhibited a broad minimum that is indicative of disorder as the main form of secondary structure. The presence of a slight shoulder in the spectra indicated some helical content, but whether this helix is transiently formed or stable—as well as where helixes occur in the chain—requires further study. Additionally, we found that MSL10^N^ exhibited helical characteristics in the presence of TFE and the small molecule glycerol and self‐associated in high temperature and PEG‐induced crowding (Figure 2b–f). Responses to crowding and osmolyte changes in vitro via folding and self‐association have been documented in the seed‐expressed intrinsically disordered proteins LEA and FLOE1 (Artur et al., 2019; Dorone et al., 2021; Koubaa et al., 2019; Rivera‐Najera et al., 2014). Some instances of environmentally driven folding are also seen in the disordered ASR proteins, which are associated with abscisic acid signaling during fruit ripening and drought response (Hamdi et al., 2017). MSL10^N^ now provides another example of an intrinsically disordered region from plants that is susceptible to conformational changes in response to physiochemical environmental modulations in vitro. Future studies are needed to determine if these traits are relevant in vivo.

Conserved motifs within IDRs often serve as modules of protein interaction (Ahrens et al., 2017). Molecular recognition features (MoRFs) are small 5–25 residue sequences that transition from a disordered to an ordered state upon interacting with a binding partner. They are found almost exclusively within IDRs (Mohan et al., 2006; Yang et al., 2019) and have demonstrated importance in some binding events (Mohan et al., 2006). The helical folding of MSL10^N^ in response to TFE and glycerol (Figure 2c,f) suggests that the N‐terminus could participate in such disorder‐to‐order‐inducing interactions. The disordered binding region predictor ANCHOR2 (Mészáros et al., 2018) predicted that the first 60 residues of MSL10^N^ and residues 70–95 comprise protein binding regions and MoRFPred and MoRFChibi–Web tools (Disfani et al., 2012; Malhis et al., 2016) predicted MoRFs at amino acids 1–33, 74–91, and 134–145 (Figure S5a). These sequences are conserved among all MSL9 and MSL10 orthologs investigated (Figure S5b), constituting some of the most conserved sequences within the otherwise poorly conserved N‐termini. In addition, all three predicted MoRF regions in Arabidopsis MSL10^N^ either contained or were adjacent to at least one N‐terminal phosphorylation site previously identified by proteomic analyses (S29, S57, S128, S131, and T136) (summarized in Veley et al., 2014) (Figure 3). This is of interest as phosphorylation may act as a key regulatory factor in MoRF transitions and function (Darling & Uversky, 2018; Mohan et al., 2006; Wright & Dyson, 2015).

However, our results indicated that phosphorylation does not cause the folding of MSL10^N^ (Figure 3). Instead, phosphorylation may regulate specific protein interactions by mediating or blocking the recruitment of that binding partner (Sridharan et al., 2022; Zhuang et al., 2009). Additionally, phosphorylation can change the characteristics of the disordered global ensemble that an IDR exhibits (Martin et al., 2016). As disordered regions rapidly sample myriad different conformations—with some conformations being sampled at a higher propensity than others—changing the charged state of the domain may modify the types of conformations allowed in the ensemble. Changes in this global ensemble can then produce newly favorable interactions with particular binding partners.

### 3.2 The N‐termini of MSL8, MSL9, and MSL10 show distinct self‐assembly characteristics in vitro

Purified recombinant MSL8^N^, MSL9^N^, and MSL10^N^ undergo self‐assembly in notably different physiochemical environments in vitro (Figures 4 and 5). Although MSL10^N^ did not exhibit self‐assembly under many tested conditions, it did form small, clustered condensates when introduced to environments with higher molecular crowding (Figures 2e and 4e). Conversely, MSL9^N^ spontaneously formed condensates in low salt conditions (Figure 4b–d). The addition of PEG 400 decreased condensate size at a given concentration of salt, implying that crowding reduces the propensity of MSL9^N^ to condense (Figure 4e). We note that molecular crowding agents such as PEG have pleiotropic effects that may be responsible for some of the behavior observed here (Christiansen et al., 2013; Zhou et al., 2008). MSL9^N^ also showed decreased self‐assembly into condensates at low temperatures (Figure 5d). MSL8^N^ formed assemblies consisting of small spherical condensates that resemble condensing systems that have reduced dynamics (Wang et al., 2018; Zhang et al., 2015). These condensates formed in low salt, at lower protein concentrations than MSL9^N^, and—opposite to MSL9^N^ condensation—were enhanced at decreased temperatures (Figure 5). Condensates formed by MSL8^N^ and MSL10^N^ are spherical but appear to be less dynamic than MSL9^N^ since they are smaller and they cluster into assemblies. These assemblies somewhat resemble amorphous protein aggregates, which form when misfolded or improperly folded globular proteins are denatured (e.g., at high temperatures) driving abberant interactions among unburied hydrophobic residues (Alberti et al., 2018). However, the assemblies that we observed are composed of small, consistently sized spherical bodies which are morphologically distinct from amorphous aggregates and more closely resemble bona fide condensing systems (Zhang et al., 2015). Furthermore, the IDRs examined in this study are largely devoid of hydrophobic residues and cluster more as temperatures decrease, altogether suggesting the absence of denaturing‐induced aggregation. To summarize, all three N‐terminal domains tested here exhibited distinct characteristics of self‐assembly, and these distinctions are likely sequence‐encoded.

As noted above, MSL8^N^, MSL9^N^, and MSL10^N^ show little sequence conservation despite evolutionary relatedness and conserved characteristics such as intrinsic disorder, hydrophobicity, and charge segregation (Figures S1 and 4a). Sequence differences may drive distinct responses to various environmental contexts. In line with the stickers and spacers model of biomolecular condensation (Emenecker et al., 2020), several charged and aromatic residues found in MSL9^N^ (R4, F31, Y60, F62, R80, F102, Y104, R108, R114, F120, F125, and R127) but absent in MSL10^N^ may serve as additional stickers and be responsible for discrepancies in assembly between the two MSL family members (Martin et al., 2020). Moreover, the longer length and enrichment of serine and glutamine residues (putative spacers) in MSL8^N^ may confer a greater propensity to form small condensates with reduced dynamics (Wang et al., 2018). Future work should target these residues as potential sequence determinants of IDR behavior.

### 3.3 Potential relevance of MSL^N^ assembly in vivo

Intrinsic disorder in membrane proteins has the potential to serve multiple regulatory functions. Self‐assembly into condensates could modulate protein–protein interactions or post‐translational modification (Alberti & Hyman, 2021). Phase separation at the membrane is known to contribute to membrane bending (Feng et al., 2019; Yuan et al., 2021) and could serve to modulate the activation of MS ion channels through changes in membrane biophysics. Future studies will determine if such potential IDR functions are relevant to MSL10's physiological function during cell swelling in seedlings (Basu & Haswell, 2020), MSL8's function during pollen grain hydration or germination (Hamilton et al., 2015), or the unknown functions of MSL9. Regardless of potential biological functions, our findings demonstrate that evolutionarily related biomolecules can exhibit drastically different physiochemical properties—even in the presence of relatively high sequence similarity.

In summary, the findings shown here provide insight into the structural character of the N‐termini of three members of the Arabidopsis MSL family of mechanosensitive ion channels, MSL8, MSL9, and MSL10. Each of the three channels studied here has different expression patterns in the plant and is involved in distinct functions within those tissues; they also have very different N‐terminal IDR sequences that exhibit distinct behaviors. This study sets the stage for research into the relationship between in vitro MSL IDR behavior and in vivo function.

## 4 METHODS

### 4.1 Computational analyses

Predictions of intrinsic disorder were performed using several webserver‐based predictors, including IUPred2A (https://iupred2a.elte.hu/), FoldIndex (https://fold.weizmann.ac.il/fldbin/findex), PONDR‐VLXT (http://www.pondr.com/), SPOT‐Disorder2 (https://sparks-lab.org/server/spot-disorder2/), PrDOS (http://prdos.hgc.jp/cgi-bin/top.cgi), CSpritz (http://old.protein.bio.unipd.it/cspritz/), PONDR‐FIT (http://original.disprot.org/pondr-fit.php), DISOPRED3 (http://bioinf.cs.ucl.ac.uk/psipred/), and MFDp2 (http://biomine.cs.vcu.edu/servers/MFDp2/). Protein binding region and MoRF predictions were made using the ANCHOR2 (https://iupred2a.elte.hu/), MoRFPred (http://biomine.cs.vcu.edu/servers/MoRFpred/), and MoRFChibi_Web (https://morf.msl.ubc.ca/index.xhtml) algorithms. The PredictProtein (https://predictprotein.org/) and Composition Profiler (http://www.cprofiler.org/cgi-bin/profiler.cgi) webservers were used for analysis of the amino acid composition of the MSL N‐termini. Topology predictions were performed using the TMHMM 2.0 (http://www.cbs.dtu.dk/services/TMHMM/) and ARAMEMNON webservers (http://aramemnon.uni-koeln.de/). The CIDER (http://pappulab.wustl.edu/CIDER/) webserver was used for Das‐Pappu plot generation and calculation of κ‐values (Holehouse et al., 2017). To compare the global propensities of disorder of the wild‐type, phosphodead, and phosphomimetic variants of the MSL10 N‐terminus, disorder scores for all residues of each N‐terminal variant (residues 1–164) were averaged and reported for five different prediction algorithms.

### 4.2 Alignments and aligned disorder scoring

MSL8, MSL9, and MSL10 sequences were aligned using the blastp algorithm in the BLAST webserver (Altschul et al., 1990); one position was manually corrected (gap shifted at aligned positions 144–149). The ClustalW program embedded within the MEGA7 software package was used to align *A. thaliana* MSL10 and putative plant orthologs reported in (Basu et al., 2020), (pairwise alignment—gap opening penalty = 15 and gap extension penalty = .5; multiple alignment—gap opening penalty = 15 and gap extension penalty = .2; residue‐specific penalties and hydrophilic penalties ON; gap separation difference = 4). To plot the disorder scores of MSL8 and MSL9 and to find the median IUPRED2A disorder score of all MSL9 and MSL10 orthologs, the process described in Christensen et al. (2019) was applied. Briefly, at an aligned position in *A. thaliana* MSL10, the alignment of *A. thaliana* MSL10 with each individual ortholog was manually compared, and nonaligning regions were removed from consideration. The disorder scores for all ortholog residues that aligned with a position of MSL10 were medialized and reported for that aligned position.

### 4.3 MSL9^N^ and MSL10^N^ protein expression and purification

*E. coli* strain DE3 (Rosetta) chemically competent cells were transformed with pET‐26b(+) plasmids containing the protein domain of interest. Overnight cultures were diluted into sterilized LB media and incubated with shaking at 37°C until reaching an optical density at 600 nm (OD_600_) of approximately .5; 1 mM isopropyl β‐d‐thiogalactopyranoside (IPTG) was added to the grown cultures, and protein induction was carried out at 37°C with shaking for 2 h. Cultured cells were flash frozen prior to storage at −80°C.

For purification, each pellet was thawed over ice and resuspended in 50 mL of lysis buffer (50 mM sodium phosphate, 1 mg/mL lysozyme, .25% Tween 20, 300 mM NaCl, and 10 mM imidazole; supplemented with .1 mM PMSF, 3 μM Leupeptin, and 1 μM Pepstatin). Resuspended pellets were incubated on ice for 30 min and then sonicated 5–10 times (1 s pulses, 50% amplitude, 10 s timer) until pale yellow in color, and debris was spun down. The resulting supernatant was added to tubes containing HisPur NiNTA resin (Thermo Fisher Scientific) and tubes were rocked for 30 min at 4°C. The supernatant was removed, and the resin was washed three times. The protein was eluted with elution buffer (50 mM sodium phosphate, 300 mM NaCl, 250 mM imidazole). Eluates were stored at 4°C overnight before further sample preparation steps took place. For eluates placed in long‐term storage, 50% glycerol was added to eluates to achieve a final sample containing 10% glycerol before flash freezing in liquid nitrogen and storing at −80°C.

### 4.4 Far‐UV CD sample preparation and measurements

Ni‐NTA eluates were thawed on ice and desalted via the PD‐10 desalting column system (GE Healthcare) following the manufacturer's gravity flow protocol. Following initial desalting, samples were subjected to Pierce PES 2–6 mL concentrator columns (Thermo Fisher Scientific) to further desalt the sample, concentrate the protein to 1–2 mg/mL, and buffer swap into 20 mM sodium phosphate buffer (pH = 7.4). Final sample purities and concentrations were estimated with Coomassie staining of SDS‐PAGE gels, Bradford assays, and UV absorbance measurements at 280 nm performed in triplicate on a Nanodrop One Microvolume UV–Vis Spectrophotometer (Thermo Fisher Scientific).

CD was performed using the JASCO J‐810 CD Spectrometer set at 20°C. A .1 cm path length cuvette was measured over the range of 190–260 nm. His‐tagged protein samples were used in the concentration range of .1–.3 mg/mL protein, as estimated by Bradford Assay. Six accumulations were taken and averaged for each measurement. For variable temperature CD, a single aliquot of purified MSL10 N‐terminus was exposed to progressively increasing temperatures, from 10°C to 80°C. For conditional CD experiments (including TFE, PEG 400, and glycerol treatments), measurements made at each percentage of a given treatment were conducted with a separate aliquot of purified protein. As proposed by Bruch et al. (1991), the ratio of values found at 208 and 222 nm was calculated to assess the character of alpha‐helical content present within the analyzed protein.

### 4.5 Sample preparation and microscopy for in vitro condensate imaging for MSL9 and MSL10

Size exclusion chromatography was performed on a HiLoad 16/600 Superdex 200 pg (GE Life Sciences) connected to an AKTA FPLC (GE Life Sciences) to further purify samples isolated via His‐tag purification and buffer swap into a “high salt” buffer (200 mM NaCl, 20 mM sodium phosphate, pH = 7.5). The concentrations and purities of fractions were assessed via absorbance at 280 nm and Coomassie staining, respectively, before pooling desired fractions and concentrating with Pierce PES 2–6 mL concentrator columns (Thermo Fisher Scientific). Concentrated protein was aliquoted and flash frozen in liquid N_2_ before storage at −80°C.

For imaging, His‐tagged protein was labeled with Alexa Fluor 488 NHS Ester (Thermo Fisher Scientific) per the manufacturer's recommendations. Labeled and unlabeled variants of a given His‐tagged protein were mixed in a molar ratio of 1:400. In a 1.6‐mm‐deep silicon isolator well (Grace Bio‐Labs) adhered to a standard microscope slide, protein in 200 mM NaCl, 20 mM sodium phosphate, pH = 7.5 was diluted with 20 mM sodium phosphate (with some concentration of NaCl, if required) to achieve the desired combination of NaCl and protein concentration. Similarly, samples prepared in 20 mM sodium phosphate were mixed with PEG 400 to achieve a final PEG concentration of 30%. Mounted samples were then imaged immediately using the 20X objective of an Olympus IX83 confocal microscope at room temperature. Image brightness and contrast were adjusted (equivalently for all images) in post to improve the visibility of condensates in a monochromatic color scheme.

### 4.6 MSL8^N^ expression and purification

*E. coli* strain DE3 (Rosetta) chemically competent cells were transformed with pET‐26b(+) plasmids containing the protein domain of interest (MSL8^N^ or MSL9^N^). Overnight cultures were diluted into sterilized LB media and incubated with shaking at 37°C until reaching an optical density at 600 nm (OD_600_) of approximately .5; 1 mM IPTG was added to the grown cultures, and protein induction was carried out at 37°C with shaking for 2 h. Cultured cells were flash frozen prior to storage at −80°C. For purification, each pellet was thawed over ice and resuspended in 50 mL of lysis buffer (50 mM sodium phosphate, 1 mg/mL lysozyme, .25% Tween 20, 300 mM NaCl, and 10 mM imidazole; supplemented with .1 mM PMSF, 3 μM Leupeptin, and 1 μM Pepstatin). Resuspended pellets were incubated on ice for 30 min and then sonicated 5–10 times (1 s pulses, 50% amplitude, 10 s timer) until pale yellow in color, and the remaining pellet was spun down.

For MSL8^N^, an insoluble preparation was performed on this pellet (see below). For MSL9^N^, the resulting supernatant was added to tubes containing HisPur NiNTA resin (Thermo Fisher Scientific) and tubes were rocked for 30 min at 4°C. The supernatant was removed, and the resin was washed three times. The protein was eluted with elution buffer (50 mM sodium phosphate, 300 mM NaCl, 250 mM imidazole). The pellet containing MSL8^N^ inclusion bodies was solubilized into a resuspension buffer containing 6 M GdmCl, 20 mM Tris, and 15 mM imidazole (pH 7.5) using sonication (3 times, 1 s on and 2 s off, 30% amplitude, 1 min timer). The resulting suspension was then clarified via centrifugation (35,000 × ***g*** for 45 min). The lysate was applied to HisPur NiNTA resin (Thermo Fisher; Cat#88221) in a gravity column for 10 min, the solution was then collected and run through again before saving it as “flow through.” The column was washed with buffer containing 20 mM Tris, 30 mM imidazole, and 4 M urea (pH 7.5) until protein was no longer detected via blue coloration in Bradford reagent droplets. Protein was eluted using an elution buffer containing 20 mM Tris, 350 mM imidazole, and 4 M urea (pH 7.5). All eluates were stored at 4°C overnight before further sample preparation steps took place. For eluates placed in long‐term storage, 50% glycerol was added to eluates to achieve a final sample containing 10% glycerol before flash freezing in liquid nitrogen and storing at −80°C. The purities of fractions were assessed via Coomassie staining before pooling desired fractions and concentrating with .5 mL Peirce PES concentrator columns (Thermo Fisher; Cat#88513). For unlabeled protein, samples were buffer swapped into 200 mM NaCl and 20 mM sodium phosphate using .5 mL Zeba spin desalting columns (Thermo Fisher; Cat#89882) immediately before imaging.

### 4.7 MSL8^N^ in vitro condensate imaging

For labeled protein samples, both MSL8^N^ and MSL9^N^ were placed in a high salt storage buffer (1 M NaCl, 200 mM sodium phosphate) using gravity PD‐10 desalting columns (GE Healthcare). The protein was labeled with Alexa Fluor 647 NHS (Invitrogen; Cat#A37573) per the manufacturer's recommendations. Any MSL8^N^ condensates that formed in the cold during labeling were spun down and removed. Labeled protein was aliquoted before flash‐freezing and storing at −80°C. Labeled and unlabeled variants of a given His‐tagged protein were mixed in a molar ratio of 1:400. In a 1.7‐mm‐deep silicon isolator well (Grace Bio‐Labs; SKU#665201) adhered to a standard microscope slide, protein in 200 mM NaCl, 20 mM sodium phosphate, pH = 7.5 was diluted with 20 mM sodium phosphate to achieve the desired combination of NaCl and protein concentration. Mounted samples were then imaged immediately using the 20X objective of a Nikon T2i microscope confocal microscope at room temperature or with an added ice pack to cool the sample to 4°C. Image brightness and contrast were adjusted (equivalently for all images) to improve the visibility of condensates.

### 4.8 GenBank accessions

The sequences for MSL8 (At2G17010.1), MSL9 (AT5G19520.1), and MSL10 (AT5G12080.1) can be found on The Arabidopsis Information Resource (TAIR). MSL9 and MSL10 orthologs used for predictions and alignments can be found at the following GenBank accession numbers: *A. thaliana*—NP_196769.1; *Arabidopsis lyrata*—XP_002873549.1; *Brassica rapa*—XP_009121883.1; *Brassica napus*—XP_013676093.1; *Camelina sativa*—XP_010453270.1; *Medicago truncatula*—XP_024633438.1; *Vitis vinifera*—XP_002279755.1; *Solanum lycopersicum*—XP_004245056.1; *Solanum tuberosum* XP_006350354.1; *Zea mays*—XP_008649202.1; *Oryza sativa –* XP_015641284.1; *Sorghum bicolor*—XP_002438025.1; *Setaria italica*—XP_004964936.1; *Brachypodium distachyon*—XP_003560953.1.

## AUTHOR CONTRIBUTIONS

**Aidan J. Flynn:** Conceptualization; investigation; and writing; **Kari Miller:** Conceptualization; investigation; and writing; **Jennette M. Codjoe:** Conceptualization; writing; and supervision; **Matthew R. King:** Conceptualization; writing; and supervision; **Elizabeth S. Haswell:** Conceptualization; writing; supervision, and project management.

## CONFLICT OF INTEREST STATEMENT

The Authors did not report any conflict of interest.

## PEER REVIEW

The peer review history for this article is available in the Supporting Information for this article.

## Supporting information


**Supporting Information S1.** Peer Review

**Data S2.** Supporting Information.
