# Supplementary material for: Mechanosensitive ion channels MSL8, MSL9, and MSL10 have environmentally sensitive intrinsically disordered regions with distinct biophysical characteristics in vitro
Source: Plant Direct. 2023 Aug 3;7(8):e515. doi: 10.1002/pld3.515 (PMC10400277; doi:10.1002/pld3.515)
Supplement: Supplementary file 2 — Data S2. Supporting Information. [file PLD3-7-e515-s001.pdf]

```
               *                                        *             *
AtMSL10   1    MAEQKSSNGGGGGGDVVINVPVEE-ASRRSKE------MASP------------ES---------
AtMSL9    1    MAERRVSNGE----EVVINVSDKE-DSKDPRASPSFNPLASP------------DS---------
AtMSL8    1    MDFRNSFKSH----SSYKQIRSPGDQSEPSPEHLPILHDHHPDHSGMVVDDQKPDSTRSSLDDGR

AtMSL10   38   -----------------------------------------------------------------
AtMSL9    40   -----------------------------------------------------------------
AtMSL8    62   NAPVERDASYKFQDNTTGTSTDHTAVRTSDKDPIAISRKGDRLSGSFDFVHGKLPVDESPTKMVA

AtMSL10   38   --------------EKGVPFSK------SPSPEISKLVGSPNKPPRAPNQNNVGLTQRKSFARSV
AtMSL9    40   --------------DAGIEKSKPVPPISIPTPEIYKFSGSVHKPPKIPSPE--GLVRRKSLSRSI
AtMSL8    127  GEPVNRQWRGRNNEEITLDVDQENDDVSHQTMPTPTSTARTSFDASREMRVSFNVRRAGGAFVAG
                    *                       *
AtMSL10   83   YSKPKSRFVDPSCPVDTSILEEE----VREQLGAG----FSFSRASPNNKSNRSVGSPAPVTPSK
AtMSL9    89   YSKPKSRFGEQQSFRYDSTREENGGRSLREQFGAGSFARGSFDRASPNNKSNRSVAS---AALSK
AtMSL8    192  SVPSSSSHSSSSSSATMRTNQDQPQLQE-EEVVRCTSNMSFQRKSELISRVKTRSRLQDPPREEE
                                      *
AtMSL10   140  VV----------------VEK-DEDEEIYKKVKLNREMRSKI
AtMSL9    151  VA----------------EEEPDENEEIYKKVKLHRVKRSGM
AtMSL8    256  T-PYSGWRSGQLKSGLLA-DIDEEDDPLAEEDVPDEYKRGKL
```

Figure S1. Alignment of *Arabidopsis thaliana* MSL8, MSL9, and MSL10 N-terminal protein sequences. Positions with identical residues are indicated with asterisks. Hydrophobic amino acids are in black, acidic residues in red, basic residues in blue, aromatic residues in orange, polar residues in green, and proline in pink.

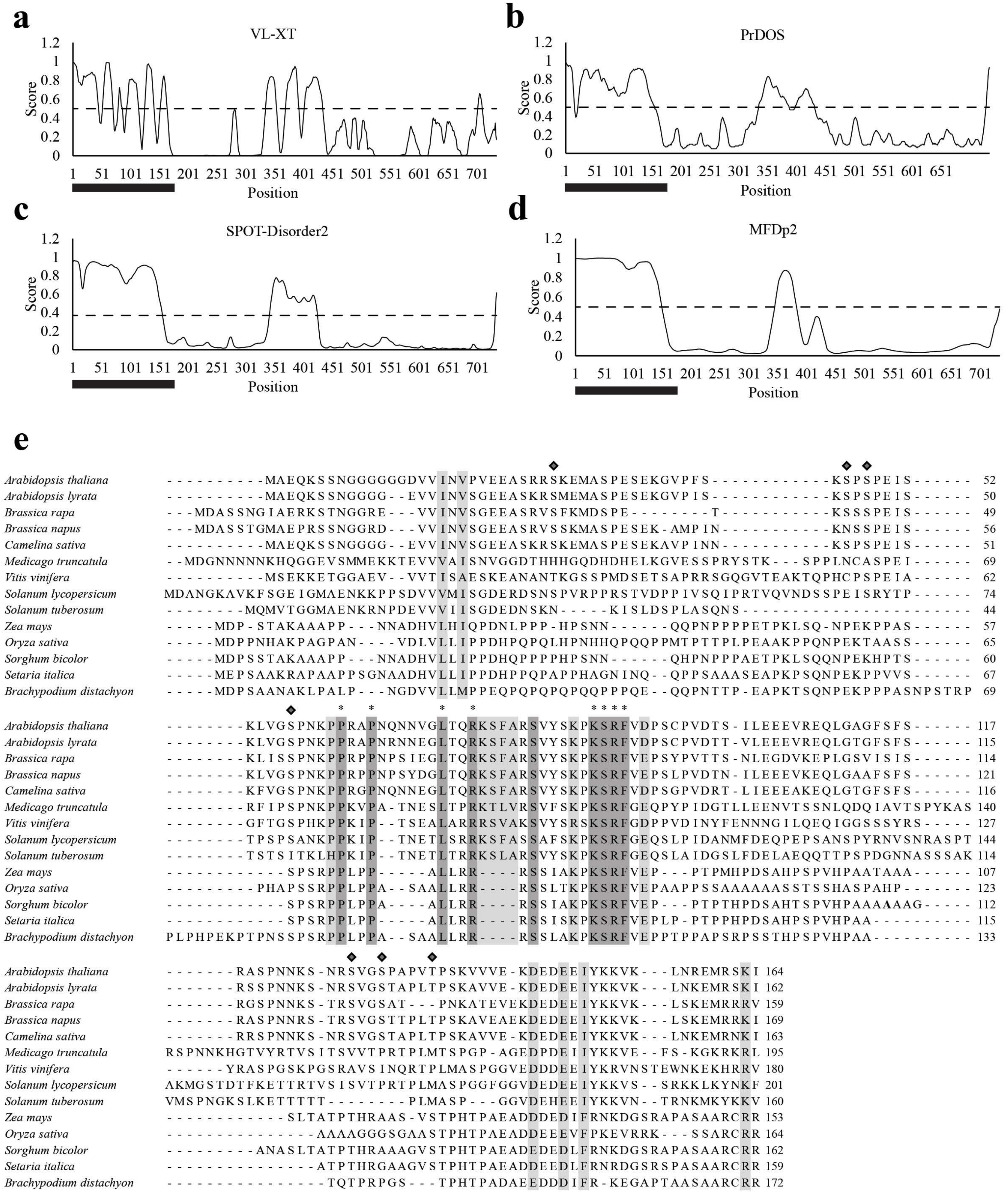

Figure S2. The N-termini of MSLs are predicted to be disordered. (a-d) Predicted disorder profiles for full-length Arabidopsis MSL10. Residues with a calculated disorder propensity greater than a given recommended algorithm threshold are predicted to be part of a disordered regions, indicated by dashed black horizontal lines. Black bar indicates the N-terminal domain of MSL10. (e) Alignment of the N-terminal sequence of *Arabidopsis thaliana* MSL10 with 13 putative orthologs. Positions with identical residues are designated by dark shading and asterisks, while positions with conserved properties are designated by light shading. Diamonds indicate the seven phosphorylation sites in the MSL10 N-terminus.

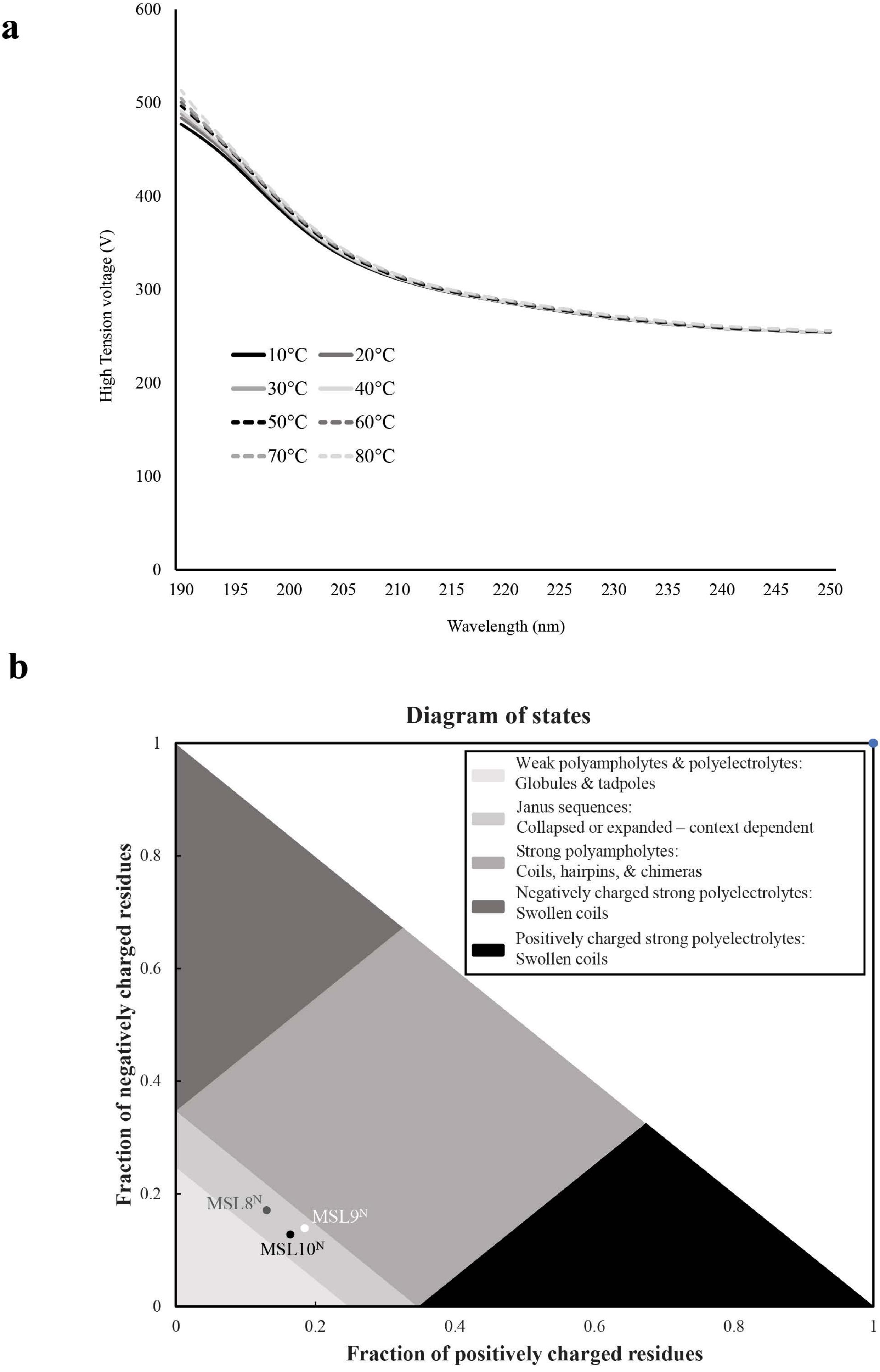

Figure S3. (a) High tension voltage curves associated with MSL10^{N} with increasing temperature. Measurements were obtained with 0.175 mg/mL protein in 20 mM sodium phosphate buffer, pH 7.4. (b) Positioning of MSL8^{N}, MSL9^{N}, and MSL10^{N} on a Das-Pappu plot generated using the CIDER webtool.

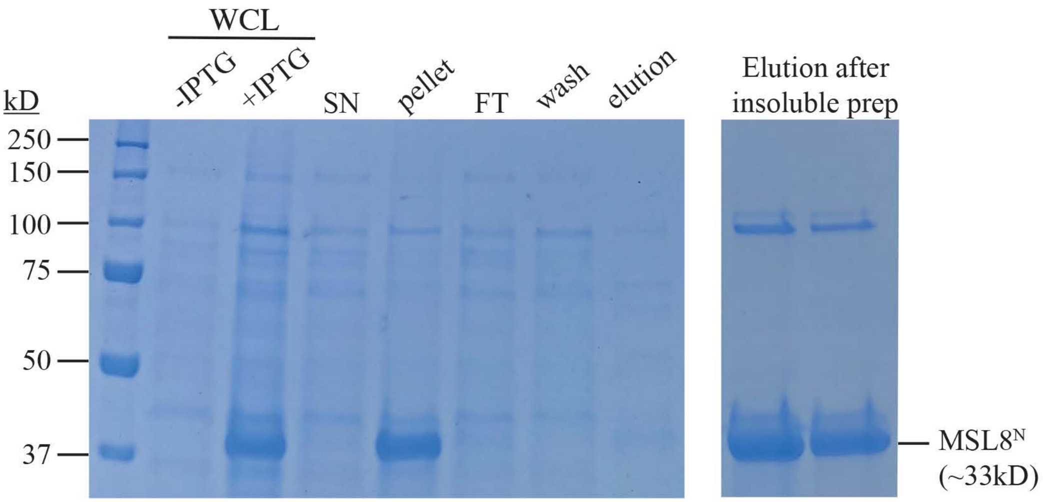

Figure S4. Coomassie-stained SDS-PAGE gel of samples taken during two MSL8^N purifications; soluble (left) and insoluble (right). WCL, whole cell lysate; SN, supernatent; FT, flow-through.

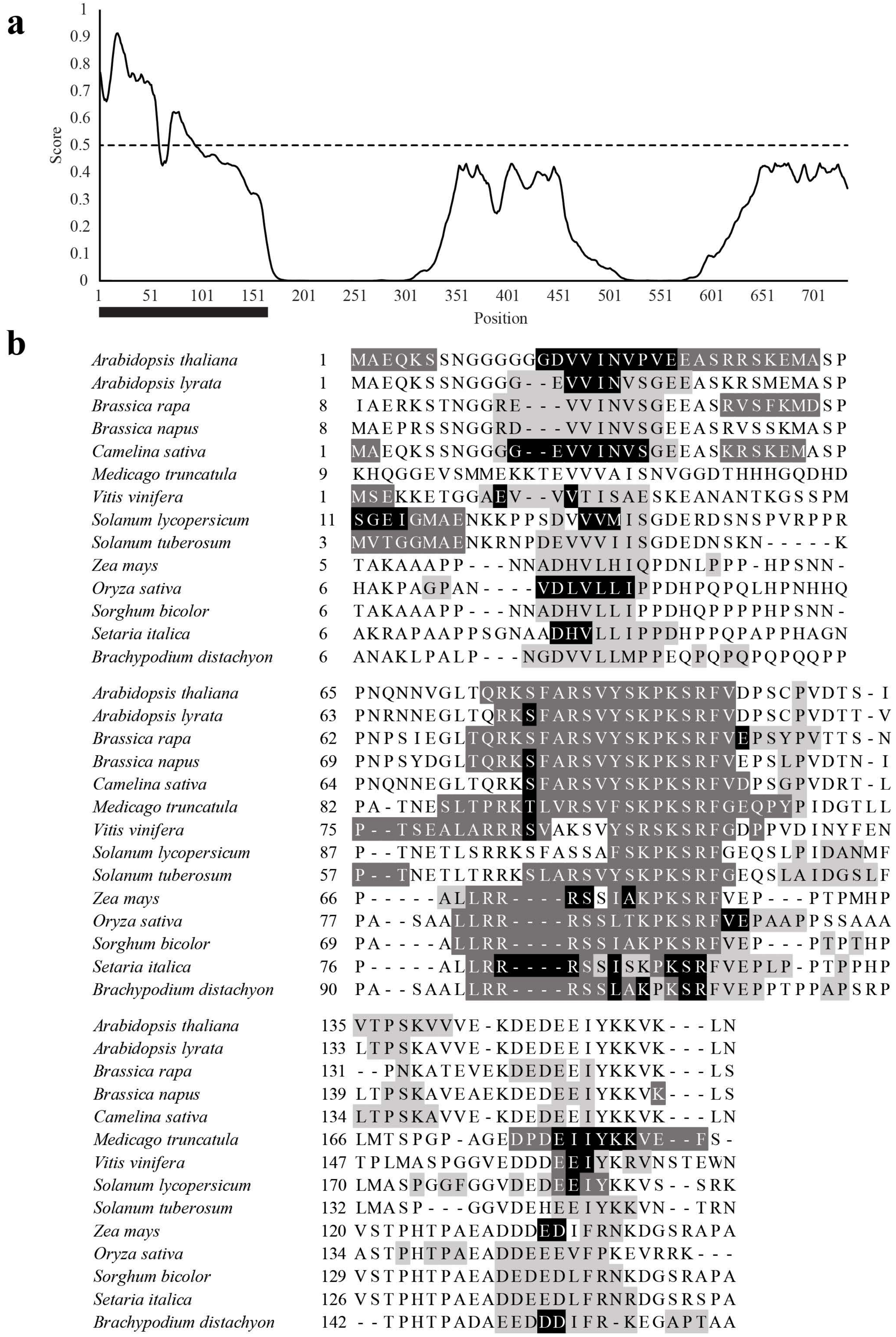

Figure S5. Predicted sites of protein interaction in the N-terminus of MSL10 and putative orthologs. (a) ANCHOR2 profile for full-length MSL10. Sequences assigned a score greater than 0.5 are predicted to be protein binding regions, as indicated by the dashed black horizontal line. The black bar indicates the residues of the MSL10 N-terminus. (b) MoRF regions identified by the MoRFPred and MoRFChibi_Web webservers for *Arabidopsis thaliana* MSL10 and 13 putative MSL10 orthologs. Amino acids predicted by MoRFPred (P > 0.5) are highlighted in light grey and MoRFChibi_Web (MCW > 0.7) predictions are highlighted in dark grey. Amino acids identified as potential MoRF residues by both MoRFPred and MoRFChibi_Web are highlighted in black.
