# Supplementary material for: Mechanosensitive ion channels MSL8, MSL9, and MSL10 have environmentally sensitive intrinsically disordered regions with distinct biophysical characteristics in vitro
Source: Plant Direct. 2023 Aug 3;7(8):e515. doi: 10.1002/pld3.515 (PMC10400277; doi:10.1002/pld3.515)
Supplement: Supplementary file 1 — Supporting Information S1. Peer Review [file PLD3-7-e515-s002.docx]

**DECISION LETTERS – Round 1**

April 25, 2023

Elizabeth S. Haswell
Washington University in St Louis
St Louis, Missouri 63130


RE: Mechanosensitive ion channels MSL8, MSL9, and MSL10 have environmentally sensitive intrinsically disordered regions with distinct biophysical characteristics

Dear Dr. Haswell:

Thank you for submitting to Plant Direct. All required reviews have been returned and we have now finished our evaluation of your manuscript. In light of the reviewers' and editor's comments, further revisions are needed before the paper can be accepted for publication in Plant Direct.

Please view the reviewers' comments below and use their suggestions as a guide while you work on your revision.

When uploading the revised version of this article, please be sure to include the following:

-A word document that contains your response to the reviewers. You should respond to each reviewer comment and note the changes made to the manuscript. If you do not agree with a reviewer's comment and choose not to make a suggested revision, please explain why. Please try to provide as complete an answer as possible to each reviewer's criticisms.
-A tracked changes document with each change highlighted
- A clean version of the latest version of the manuscript

To upload your revision, please click the link below.
https://plantdirect.msubmit.net/cgi-bin/main.plex?el=A2Lr2Zn1A3FFH2I1A9ftdhUeEgYzBWsj5B8eaEHsLgZ

In order to provide as timely a service as possible, we ask that your revision is resubmitted within three months after receipt of this request. If an extension is needed, please send a request, along with a brief explanation, to the editorial office at plantdirect@wiley.com .

Please note that, in addition to publishing reviewer comments, the author's responses to review comments will also be published alongside the final version of the paper. If you would not like the author's responses to be published, please contact the editorial office at plantdirect@wiley.com .


Thank you very much for giving us an opportunity to review your work. I look forward to receiving the next version.


Sincerely,

Chi Zhang

Hsou-min Li

Editor, Plant Direct


---------------------------------------------------------------------------- Reviewer comments:
Reviewer #1:

In this manuscript, the authors predicted the IDRs in MSL8, MSL9, and MSL10 and analyzed the intrinsic disorder in N-terminus using some in vitro approaches. But further in vivo evidences are essential. Because recombinant HIS-tagged N-terminus of MSL can't represent the full-length MSL and the real in vivo IDR characteristic of MSL in plants. Suggest the authors to perform such as in vivo localization assay and FRAP assay in plants.

1. Result 1 and 2 can be merged into result 1. They both belong to the prediction of IDRs in MSL8, MSL9, and MSL10.
2. Fig 2A. which band is N-terminus of MSL10?
3. Result: Phosphomimic and phosphodead mutations do not affect folding of MSL10N.
In this part, the authors can't use recombinant phosphodead mutations to test the influence of phosphorylation on IDR. Because E. coli may lack the kinase (from the plant) for the phosphorylation of MSL10. The method is wrong.
Even recombinant N-terminus of MSL10 was phosphorylated in E.coli, the phosphorylation status may be different from that in vivo in plants.
4. Fig S4. The upper band about 99 kDa might be just the unspecific bands. Did the author perform the Western blot to confirm that binds from HIS-MSL8N?


Reviewer #2:

All in all, the subject is novel but there are some issues needing to be solved. See my review document for details.

The authors identified and characterized intrinsic disorder (IDR) in the soluble cytoplasmic N-terminal domains of MSL8, MSL9, and MSL10, three mechanosensitive ion channels. Bioinformatic tools uniformly predicted that the cytosolic N-termini of MSL channels are intrinsically disordered. The authors also tested the response of MSL10N under a series of environmental stimuli: MSL10N is largely disordered and may experience a modest increase in homotypic interactions with elevated temperature; MSL10N is capable of folding in the presence of TFE in vitro; the disordered MSL10N retains the ability to fold and/or self-associate in various solution environments; MSL10N is largely disordered regardless of the phosphorylation state. Finally, they found MSL8N, MSL9N, and MSL10N have sharply differing propensities for self-assembly into condensates. The finding that the presence of IDRs in the soluble N-terminus of Arabidopsis thaliana mechanosensitive ion channels is novel. However, some issues need to be addressed.
1. To support that MSL8N, MSL9N and MSL10N are IDR proteins, the authors conducted both bioinformatics and experimental studies. However, both negative control (non-IDRs) and positive control (known IDRs) should be involved at each step of validation.
2. The authors mentioned that MSL8N, MSL9N, and MSL10N contain 46-58% disorder-promoting residues, and only 35-37% order-promoting residues. What are the proportions of disorder-promoting and order-promoting residues in canonical IDR proteins? The authors need to compare
MSL8N,MSL9N, and MSL10N with the canonical IDR proteins in terms of these proportions to test if disorder-promoting residues are more or less enriched in MSL8N,MSL9N, and MSL10N.
3. The authors predicted the propensity of MSL8N,MSL9N, and MSL10N for the disorder. The authors need to randomly select a set of known IDRs as a background to evaluate the propensity of MSL8N,MSL9N, and MSL10N.
4. The authors stated that TFE induces structure in disordered regions with susceptibility to forming alpha-helices. Is this a feature that is typical structure of IDR proteins? The authors need to use some known IDR proteins that have similar features under the same concentration of TEE as a positive control.
5. The authors mentioned "Previous in vitro studies have demonstrated changes in plant IDR structure in response to molecular crowders" and tested the response of MSL10N to polyethylene glycol 400 (PEG 400) and glycerol. How did other plant IDR proteins respond to molecular crowders? Do those plant IDR proteins have the same or similar the response to crowders like the response of MSL10N to polyethylene glycol 400 (PEG 400) and glycerol? The authors need to compare the response of known plant IDRs and MSL10N to the same molecular crowders.
6. The author mentioned "Some IDRs undergo folding events in response to changes in phosphorylation state and MSL10 did not". The authors need to address why the results of MSLs are different from the previous studies of IDR proteins. The authors need to compare MSL10N with other IDRs that are also disordered regardless of the phosphorylation state. Do they have similar structures? How is MSL10N different from other IDRs that undergo folding events in response to changes in phosphorylation state?

**DECISION LETTERS – Round 2**

June 20, 2023

Elizabeth S. Haswell
Washington University in St Louis
St Louis, Missouri 63130


RE: Mechanosensitive ion channels MSL8, MSL9, and MSL10 have environmentally sensitive intrinsically disordered regions with distinct biophysical characteristics in vitro

Dear Dr. Haswell:

Thank you for submitting to Plant Direct. All required reviews have been returned and we have now finished our evaluation of your manuscript. In light of comments from reviewer #2, further revisions are needed before the paper can be accepted for publication in Plant Direct.

Please view the reviewers' comments below and use their suggestions as a guide while you work on your revision.

When uploading the revised version of this article, please be sure to include the following:

-A word document that contains your response to the reviewers. You should respond to each reviewer comment and note the changes made to the manuscript. If you do not agree with a reviewer's comment and choose not to make a suggested revision, please explain why. Please try to provide as complete an answer as possible to each reviewer's criticisms.
-A tracked changes document with each change highlighted
- A clean version of the latest version of the manuscript


To upload your revision, please click the link below.
https://plantdirect.msubmit.net/cgi-bin/main.plex?el=A1Lr6Zn3B7FFH3I5A9ftdhUeEgYzBWsj5B8eaEHsLgZ


In order to provide as timely a service as possible, we ask that your revision is resubmitted within three months after receipt of this request. If an extension is needed, please send a request, along with a brief explanation, to the editorial office at plantdirect@wiley.com .

Please note that, in addition to publishing reviewer comments, the author's responses to review comments will also be published alongside the final version of the paper. If you would not like the author's responses to be published, please contact the editorial office at plantdirect@wiley.com .


Thank you very much for giving us an opportunity to review your work. I look forward to receiving the next version.


Sincerely,

Chi Zhang

Hsou-min Li

Editor, Plant Direct


---------------------------------------------------------------------------- Reviewer comments:
Reviewer #1:

The authors addressed my questions.


Reviewer #2:

please revise your paper according my review

| **Attachment:** | [Reviewer 2 Review Attachment 1 - 06-20-2023 17:49:37](https://plantdirect.msubmit.net/pd_files/2023/05/17/00001339/01/1339_1_attachment_confidential_review_64963_r1tb17.pdf) |
| --- | --- |
| **Attachment:** | [Reviewer 2 Review Attachment 3 - 06-20-2023 17:49:37](https://plantdirect.msubmit.net/pd_files/2023/05/17/00001339/01/2_reviewer_attachment_3_1687233133.pdf) |

**AUTHORS’ RESPONSE**

***VERSION 2***

***We thank the reviewers very much for reading our manuscript and for taking the time to provide their thoughts. Below we address each comments point-by-point. Changes to the text are indicated in red. We also made the small suggested changes to Figure 2A and Supplementary Figure S4.***

Reviewer #1:

In this manuscript, the authors predicted the IDRs in MSL8, MSL9, and MSL10 and analyzed the

intrinsic disorder in N-terminus using some in vitro approaches. But further in vivo evidences are

essential. Because recombinant HIS-tagged N-terminus of MSL can't represent the full-length MSL and the real in vivo IDR characteristic of MSL in plants. Suggest the authors to perform such as in vivo localization assay and FRAP assay in plants.

***The assays mentioned above are certainly appropriate to test the in vivo relevance of IDRs. However, in this manuscript, we are focused on the unique biophysical characteristics of evolutionarily related molecules with a focus on their in vitro behavior. It is for this reason that we specifically chose Plant Direct as a sound science journal without an emphasis on perceived impact. As we state in the discussion, “future studies are needed to determine if these traits are relevant in vivo.” We also address the potential in vivo relevance in the last section, entitled “Potential relevance of MSLN assembly in vivo.” To clarify this point even further, we have added “in vitro” to the manuscript title and to relevant subheadings in the Discussion.***

Result 1 and 2 can be merged into result 1. They both belong to the prediction of IDRs in MSL8, MSL9, and MSL10.

***We removed the second results subtitle (“The N-termini of plasma membrane-localized MSL channels are predicted to be disordered”) and added “and are predicted to be disordered” to the first subheading.***

Fig 2A. which band is N-terminus of MSL10?

***Thank you for this question. We have now added a marker indicating the band that is the N-terminus of MSL10.***

Phosphomimic and phosphodead mutations do not affect folding of MSL10N. In this part, the authors can't use recombinant phosphodead mutations to test the influence of phosphorylation on IDR. Because E. coli may lack the kinase (from the plant) for the phosphorylation of MSL10. The method is wrong. Even recombinant N-terminus of MSL10 was phosphorylated in E.coli, the phosphorylation status may be different from that in vivo in plants.

***We apologize for any confusion caused by the way this section was written. The variants that were***

***purified from* E. coli *are not expected to be phosphorylated by bacterial kinases. Rather, we are making mutations to the MSL10 N-terminus that are meant to mimic phosphorylated and dephosphorylated states, a common approach in plant molecular biology. We have used these same phosphomimic and phosphodead N-terminal variants in multiple published studies (e.g. Basu et al., 2022; Codjoe et al, 2022; Basu and Haswell, 2020). To be more circumspect in our discussion of these variants, we have changed the language in several sentences within the results section “Phosphomimic and phosphodead mutations do not affect folding of MSL10N”.***

4. Fig S4. The upper band about 99 kDa might be just the unspecific bands. Did the author perform the Western blot to confirm that binds from HIS-MSL8N?

***We agree with the reviewer and have removed the speculative label for these bands.***

Reviewer #2:

To support that MSL8N, MSL9N and MSL10N are IDR proteins, the authors conducted both

bioinformatics and experimental studies. However, both negative control (non-IDRs) and positive control (known IDRs) should be involved at each step of validation.

***Please see our thoughts below in the following responses.***

The authors mentioned that MSL8N, MSL9N, and MSL10N contain 46-58% disorder-promoting

residues, and only 35-37% order-promoting residues. What are the proportions of disorder-promoting and order-promoting residues in canonical IDR proteins? The authors need to compare MSL8N,MSL9N, and MSL10N with the canonical IDR proteins in terms of these proportions to test if disorder-promoting residues are more or less enriched in MSL8N,MSL9N, and MSL10N.

***To more explicitly compare the MSL N-termini to other IDRs, we have modified the text in this section of the results to the following:***

***“Many IDRs are defined by characteristic amino acid compositions such as a depletion of hydrophobic residues that drive protein folding events and an enrichment in hydrophilic, charged, and polar residues (Uversky, 2013, 2019; Lee et al., 2014; Yachdav et al., 2014). For instance, IDRs in the DisProt database contain on average ~60% disorder-promoting residues with notably high percentages of proline (~8%), glutamate (10%), serine (~9%), and lysine (~8%). We therefore assessed the amino acid frequencies present in the soluble N-termini of MSL8, MSL9, and MSL10.”***

3. The authors predicted the propensity of MSL8N,MSL9N, and MSL10N for the disorder. The authors need to randomly select a set of known IDRs as a background to evaluate the propensity of

MSL8N,MSL9N, and MSL10N.

***While we can appreciate the reviewer’s point, it is not necessary to include IDR sets as a background to evaluate the accuracy of a disorder predictor, because these predictors were trained on a large set of IDRs and subsequently evaluated on a set of IDRs different from the training set (as well as with direct experimental assessments of structure). Furthermore, IUPRED2A was tested on multiple negative protein datasets that present different structural characteristics such as being ordered, disordered, or linker regions (See Figure S1 and other supplementary materials of https://academic.oup.com/nar/article/46/W1/W329/5026265?login=true). IUPRED2A predictions agree with many other disorder predictors that were trained on independent datasets and have been evaluated with success on the Critical Assessment of Protein Structure Prediction (CASP). IUPRED2A has received nearly 1,000 references that clearly indicate the accuracy of its predictions. Therefore, we do not believe that presenting a background IDR set is necessary to validate the predictions done here.***

4. The authors stated that TFE induces structure in disordered regions with susceptibility to forming

alpha-helices. Is this a feature that is typical structure of IDR proteins? The authors need to use some

known IDR proteins that have similar features under the same concentration of TEE as a positive control.

***Yes, this response to TFE is a feature of some IDR-containing proteins. We modified the text in the***

***Results section to more clearly communicate this:***

***“TFE induces structure in a subset of disordered regions that have a susceptibility to forming alphahelices, such as for those that fold upon protein interaction.”***

***We did not include known IDRs as positive controls for the response to TFE or crowding, because***

***identifying secondary structure in a protein region via circular dichroism is a largely independent***

***assessment – that is to say, one can evaluate the secondary structure present within a sample without a comparison to another disordered region in the same experiment (see***

***https://www.ncbi.nlm.nih.gov/pmc/articles/PMC4988895/ and https://link.springer.com/protocol/10.1007/978-1-61779-927-3_22 for examples in primary literature and reviews, respectively). There are largescale repositories of circular dichroism results such as the PCDBB (https://pcddb.cryst.bbk.ac.uk/) that clearly indicate common types of spectra observed while conducting these sorts of experiments. Many of these structural states have been confirmed by additional techniques such as NMR and crystallography. Therefore, we can qualitatively assess what sorts of structural transitions are occurring in response to a given treatment by comparing them to observed spectra for different known structural states.***

5. The authors mentioned "Previous in vitro studies have demonstrated changes in plant IDR structure in response to molecular crowders" and tested the response of MSL10N to polyethylene glycol 400 (PEG 400) and glycerol. How did other plant IDR proteins respond to molecular crowders? Do those plant IDR proteins have the same or similar the response to crowders like the response of MSL10N to polyethylene glycol 400 (PEG 400) and glycerol? The authors need to compare the response of known plant IDRs and MSL10N to the same molecular crowders.

***To better explain/address how other IDRs have been shown to respond to molecular crowders, we***

***modified the text describing these panels:***

***“Previous in vitro studies have demonstrated changes in some IDR ensembles in response to molecular crowders and small molecules. For instance, some plant IDRs such as members of the Late Embryogenesis Abundant (LEA) families take on a helical structure and undergo increased selfassociation in the presence of these components (Dorone et al., 2021; Rivera-Najera et al., 2014; Mansouri et al., 2016; Chemes et al., 2012).”***

***“Responses to crowding and osmolyte changes in vitro via folding and self-association have been***

***documented in the seed-expressed intrinsically disordered proteins LEA and FLOE1 (Rivera-Najera et al., 2014; Artur et al., 2019; Koubaa et al., 2019; Dorone et al., 2021).”***

6. The author mentioned "Some IDRs undergo folding events in response to changes in phosphorylation state and MSL10 did not ". The authors need to address why the results of MSLs are different from the previous studies of IDR proteins. The authors need to compare MSL10N with other IDRs that are also disordered regardless of the phosphorylation state. Do they have similar structures? How is MSL10N different from other IDRs that undergo folding events in response to changes in phosphorylation state?

***As we state, phosphorylation can change secondary structure ( i.e. induce an IDR to fold)—but this***

***has been only rarely documented (as in the paper we cite, Bah et al., 2015), and is far from a rule. We added a section to the Discussion to describe the many other possible mechanisms by which***

***phosphorylation may regulate function of an IDR:***

***“ . . . phosphorylation may regulate specific protein interactions by mediating or blocking the***

***recruitment of that binding partner (Zhuang et al., 2009; Sridharan et al., 2022). Additionally,***

***phosphorylation can change the characteristics of the disordered global ensemble that an IDR exhibits (Martin et al., 2016). As disordered regions rapidly sample myriad different conformations – with some conformations being sampled at a higher propensity than others – changing the charged state of the domain may modify the types of conformations allowed in the ensemble. Changes in this global ensemble can then produce newly favorable interactions with particular binding partners.”***

**DECISION LETTERS – Round 3**

June 27, 2023
Elizabeth S. Haswell
Washington University in St Louis
St Louis, Missouri 63130


MSID: 2023-01143R2
MS TITLE: Mechanosensitive ion channels MSL8, MSL9, and MSL10 have environmentally sensitive intrinsically disordered regions with distinct biophysical characteristics in vitro

Dear Dr. Elizabeth Haswell:

I am pleased to inform you that your manuscript "Mechanosensitive ion channels MSL8, MSL9, and MSL10 have environmentally sensitive intrinsically disordered regions with distinct biophysical characteristics in vitro" has been accepted for publication in Plant Direct.

Your article will appear online in the next available issue of Plant Direct. To ensure your article gets published as quickly as possible, please pay attention to the steps detailed below. We have found that most of the delays happen at this stage, especially at the payment stage, so please respond as quickly as possible when prompted.

License Agreement: Once your article has been accepted it will move to Production and undergo admin and file checking - you may receive an email with any queries we have at this stage. When all required items are received by the publisher and queries resolved, the corresponding author will receive an email from Wiley's Author Services system which will ask them to log on at https://authorservices.wiley.com/bauthor and will present them with the appropriate license for completion. Your article cannot be published until both the signed license agreement and payment of the article fee have been received.

Payment of the Open Access Article Publication Fee: All articles published in Plant Direct are fully open access: immediately and freely available to read, download and share. Plant Direct charges a publication fee to cover the publication costs. The corresponding author for this manuscript should have already received a quote from the payments team (cs-openaccess@wiley.com) with the estimated article publication fee; please email cs-openaccess@wiley.com if this has not been received. The corresponding author should log on to the Wiley Author Services site, where the publication fee can be paid by credit card or an invoice. Pro Forma can also be requested. Payment of the publication charge must be received before the article will be published online.

Proofs: You will have the opportunity to look over your paper once more when you receive the author proofs for your article. The proofs will be with you in approximately two weeks. Please note that, in addition to publishing reviewer comments, the author's responses to review comments will also be published alongside the final version of the paper. If you would not like the author's responses to be published, please contact the editorial office at plantdirect@wiley.com .

Promotion of your article: You can help your research get the attention it deserves! Check out Wiley's free Promotion Guide for best-practice recommendations for promoting your work at www.wileyauthors.com/eeo/guide. And learn more about Wiley Editing Services which offers professional video, design, and writing services to create shareable video abstracts, infographics, conference posters, lay summaries, and research news stories for your research at www.wileyauthors.com/eeo/promotion.

Thank you again for your contribution to Plant Direct. If you have any questions, feel free to contact the editorial office at plantdirect@wiley.com .

Sincerely,

Chi Zhang

Hsou-min Li

Editor, Plant Direct

**AUTHORS’ RESPONSE**

**RESPONSE TO REVIEWERS**

Reviewer #2:

please revise your paper according my review.

There are certain issues that still need to be addressed in the revised version.

1. There are some verbose sentences and paragraphs in the "Introduction" part that are not linked

to the topic and study of this paper.

Without specific examples, we find ourselves unable to address this request. Perhaps this can be

considered our preferred style of writing, and not a matter of editorial concern?

2. The research in this paper do not involve the investigation of how IDRs drive the assembly of

biomolecular condensates in response to environmental stimuli. So, there is need to give the

definitions of associative and segregate transitions.

We feel that providing the brief 3-sentence overview of different types of transitions in the

introduction is critical for including readers from disparate scientific backgrounds.

In addition, this background is important to our understanding of MSL IDR function. The stickers

and spacers model, to which the MSLs appear to adhere, was largely established with information

on these different types of transitions. Because the three MSL termini analyzed here exhibit

different sequence characteristics, the stickers and spacers that each has are likely to be slightly

different and lead to different condensate properties. These differences can be more effectively

understood by weighing the contributions of associative and segregative transitions that occur

when each of the chains experience condensation.

3. This paper does not study the mechanisms of MSL10's function. So, it is better to remove the

part about the function of MSL10, such as promoting programmed cell death.

We agree that this paper does not study the physiological function of MSL10. However, what is

described is interesting partly in light of its known functions, as we outline in the discussion.

Therefore, we prefer to keep this sentence in the introduction.
